# Mediastinal lymph node metastases in lung cancer presenting as pure ground-glass nodules: A surgical case report

**DOI:** 10.1016/j.ijscr.2020.03.021

**Published:** 2020-03-28

**Authors:** Yohei Honda, Soichi Oka, Yasuhiro Chikaishi, Masaaki Inoue, Junichi Yoshida, Daisei Yasuda

**Affiliations:** a Thoracic Surgery, Shimonoseki City Hospital, Shimonoseki, Japan; b Pathology, Shimonoseki City Hospital, Shimonoseki, Japan

**Keywords:** Pure ground-glass nodules, Multifocal ground-glass nodules, Mediastinal lymph nodes metastases, Bulla wall

## Abstract

**Introduction:**

Generally, lung cancer representing as Ground-glass nodules is associated with an early stage and good prognosis. However, we herein report a rare case of pure ground-glass nodules with mediastinal lymph node metastases.

**Presentation of case:**

A 69-year-old man underwent video assisted thoracic surgery right upper lobectomy with mediastinal lymph node dissection due to multifocal Ground-glass nodules in the right upper lobe of the lung. Histopathologically, six lung adenocarcinomas were present simultaneously. Furthermore, we detected mediastinal lymph nodes metastases that contain micropapillary component. The lung lesion containing micropapillary component was a pure Ground-glass nodule that adjoining pulmonary bulla on CT findings.

**Discussion:**

Generally, lung cancer presenting as pure ground-glass nodules is associated with an early stage and good prognosis. However, the necessity of evaluating the mediastinal lymph nodes in pure ground-glass nodules is controversial. It is reported that lung cancer adjoining the wall of a bulla tends to have a poor prognosis, even when small in size. Therefore, Ground-glass nodules with metastases might have a different pathogenesis than other nodules.

**Conclusion:**

Adenocarcinoma appearing as pure Ground-glass nodules is associated with early stage lung cancer and a good prognosis. However, the findings in our patient indicate the importance and necessity of evaluating the mediastinal lymph nodes for metastases intraoperatively.

## Highlights

• Pure ground-glass nodule with mediastinal lymph node metastases is rare.
• Micropapillary component of lung adenocarcinoma is the risk of mediastinal lymph node metastases and poor prognosis.
• The necessity of lymph node dissection of lung adenocarcinoma presenting as pure GGNs is unclear.

## 1 Introduction

Recently, the use of computed tomography (CT) for lung cancer screening and lung diseases has increased the chances of detecting ground-glass nodules (GGNs). GGNs are defined as hazy lesions <3 cm in size that do not obscure underlying bronchial structures or pulmonary vessels. Furthermore, such lesions are categorized as pure GGNs or nodules containing a solid component, collectively called part-solid nodules [1,2]. In some cases, multiple GGNs are detected, and multifocal GGNs are generally associated with atypical adenomatous hyperplasia, and early-stage lung adenocarcinoma such as adenocarcinoma in situ or minimally invasive adenocarcinoma [2]. GGNs are not usually accompanied by metastases; therefore, good treatment results are expected. However, we report a surgical case of pure, multifocal GGNs with mediastinal lymph node metastases. In this patient, we diagnosed no clinical lymph node metastases (cN0), but postoperative histopathological examination revealed mediastinal lymph node metastases (pN2). This work has been reported in line with the SCARE criteria [3].

## 2 Presentation of case

**Fig. 1:**
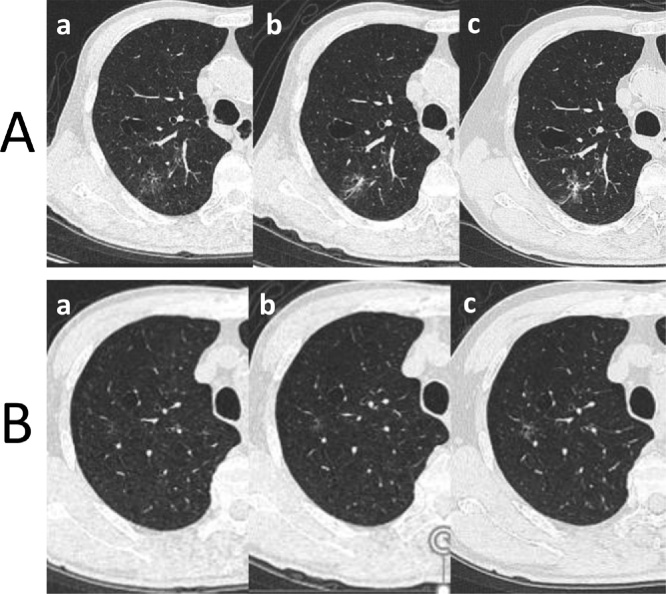
**A)** The solid component of the GGN in segment 2 had increased in size according to the CT findings. **B)** Pure GGN in segment 1 adjoining the bulla wall on CT. a. Thirteen months before surgery. b. Nine months before surgery. c. One month before surgery.

**Fig. 2:**
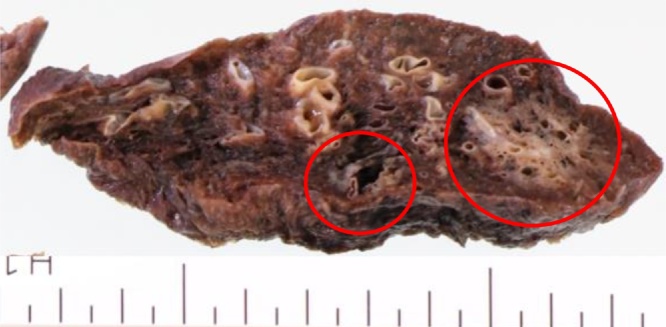
Appearance of the isolated preparation. The larger nodule measured 24 × 20 × 16 mm in size, and was yellowish-white in color and indistinct. The smaller nodule measured 15 × 15 × 12 mm in size and was also indistinct.

A 69-year-old Japanese man presented to our institute because of abnormal chest CT findings, which showed multifocal GGNs. A CT scan after 3 years of follow-up showed that one of the lesions in the right upper lobe had increased in size from 23 mm to 39 mm, and that this lesion contained a solid component 13 mm in size (Fig. 1). We did not identify swollen hilar or mediastinal lymph nodes on CT findings during follow-up, so we did not performed FDG-PET/CT. The patient had a 48-pack-year smoking history, and a history of colorectal cancer, hypertension, and diabetes mellitus. Laboratory tests showed elevated serum carcinoembryonic antigen levels. We highly suspected that the enlarged lesion in the right upper lobe was lung cancer (cT1bN0M0, stage IA2, based on the eighth edition of the International Union Against Cancer tumor-node-metastasis classification [4]). Other small GGNs that no change in size were present in the same lobe, so right upper lobectomy was considered a reasonable surgical procedure regarding radicality and the necessity of obtaining a histopathological diagnosis. Therefore, we performed right upper lobectomy with mediastinal lymph node dissection via video-assisted thoracoscopic surgery. None of the dissected mediastinal lymph nodes, which were dissected en bloc, were enlarged (Fig. 2).

**Fig. 3:**
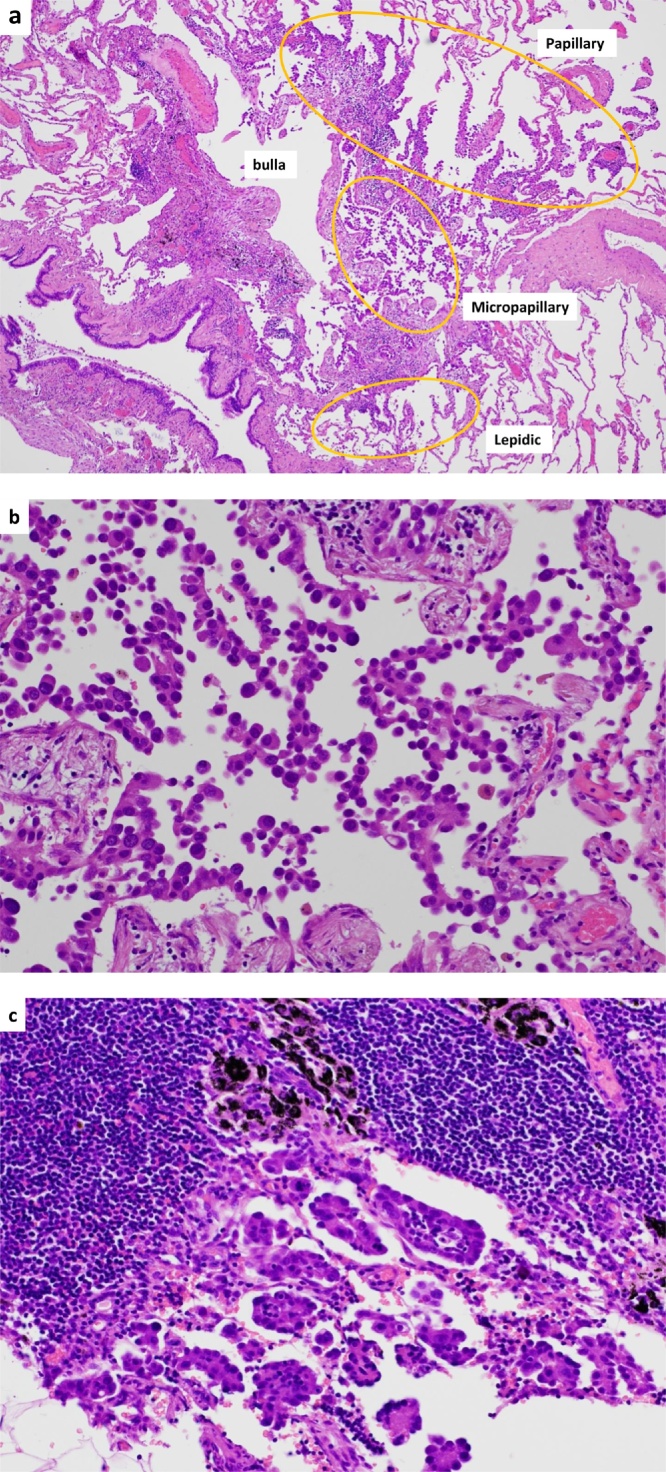
a. Overview of the invasive adenocarcinoma in segment 1. Findings included lepidic, papillary, and micropapillary adenocarcinoma. Furthermore, the micropapillary component adjoined the bulla. b. Microscopic appearance of the micropapillary component in the tumor. Tumor cells are growing in papillary tufts forming florets that lack fibrovascular cores. c. Microscopic appearance of the micropapillary component in the lymph node. Tumor cells similar to those seen in Fig. 3b are visible.

Histopathologically, six lung adenocarcinomas were present simultaneously. The enlarged tumor was invasive adenocarcinoma with an invasive size of 2.4 cm and histological subtypes of a lepidic component (75%) and a papillary component (25%), so we diagnosed pT1cN0M0, Stage.IA3. The other five tumors were Tis, T1mi, or T1a, which appeared to be early lung cancer at first glance, and the CT appearances of these tumors were pure GGNs. However, we detected mediastinal lymph node metastases (#2R and 4R), which had a micropapillary component. The primary cancer type in the metastatic lymph nodes was T1a (1.0 cm) adenocarcinoma with mixed subtypes (lepidic: 40%, papillary: 55%, micropapillary: 5%), and appeared as pure GGNs adjoining the bulla wall on CT findings. Therefore, the final pathologic staging of the pure GGN confirmed as pT1aN2M0, Stage.IIIA. The patient underwent adjuvant chemotherapy with Carboplatin (AUC = 5 mg/mL/min, on day 1) and nab-Paclitaxel (100 mg/m^2^, on days 1, 8, and 15) 7 weeks after surgery. There was no evidence of relapse 13 months after surgery.

## 3 Discussion

GGNs are detected commonly because of the widespread use of thin-section CT for lung cancer screening [5]. Lung adenocarcinomas with pure GGN appearance on CT are usually associated with an early stage and good prognosis [6]. In the JCOG0804/WJOG4507L study, Asamura et al. reported the efficacy and safety of sublobar resection for pure and part-solid GGN lung cancer. The inclusion criteria were maximum tumor size ≤2.0 cm and consolidation/tumor ratio ≤0.25. The 5-year relapse-free survival rate after sublobar resection (mainly wedge resection) was 99.7%. The authors concluded that limited resection of lung cancer matching the described criteria was standard therapy [7]. However, in the current report, we found lung adenocarcinoma with mediastinal lymph node metastases despite finding pure GGNs on CT. With pure unifocal GGN lung cancer with mediastinal lymph node metastases, we might perform sublobar resection according to the results of previous studies, such as the JCOG0804/WJOG4507L study. When we re-evaluated our patient’s CT findings, we ascertained that one GGN adjoined the bulla wall; we also found that the lymph node metastases had developed from the micropapillary component of the GGN. Emphysematous bulla are sometimes associated with lung cancer [[8], [9], [10]], so GGN with metastases might have a different pathogenesis than other nodules. Kaneda et al. reported that lung cancer adjoining the wall of a bulla tends to have a poor prognosis, even when small in size [10]. In addition, that lesion contained a micropapillary component (5% of the tumor) [10]. Yue et al. reported that a minor component of the micropapillary subtype in lung adenocarcinoma predicted lymph node metastases and a poor prognosis [11]. Prognostically, small amounts of components such as micropapillary or solid patterns may be important [12]. The findings in our patient indicate that there are sometimes highly-malignant tumors in lung adenocarcinoma that appear as GGNs, which necessitates evaluating lymph nodes for metastases.

## 4 Conclusion

We reported lung adenocarcinoma with mediastinal lymph node metastases despite the appearance of pure GGNs in CT findings. Generally, adenocarcinoma appearing as pure GGNs is associated with early stage lung cancer and a good prognosis, but the findings in our patient indicate the importance and necessity of evaluating the mediastinal lymph nodes for metastases intraoperatively in such cases. Because this is only a case report, clearer evidence from prospective studies is required to confirm that pure GGNs adjoining a bulla wall are at high risk of developing mediastinal lymph node metastases.

## Conflict of interest

The authors declare no conflicts of interest associated with this manuscript.

## Sources of funding

Junichi Yoshida received Research funding from Astellas Pharma Inc.

The other authors declare that they have no sources of funding for our research.

## Ethical approval

We got ethical approval from ethics committee of Shimonoseki city hospital, Japan.

## Consent

We had informed consent from this patient for writing this paper.

## Author contribution

Yohei Honda; study design, writing. Masaaki Inoue; study design, other. Soichi Oka; other. Yasuhiro Chikaishi; other. Junichi Yoshida; other. Daisei Yasuda; other.

## Registration of research studies

Yes, we can.

## Guarantor

Yohei Honda and Masaaki Inoue.

## Provenance and peer review

Not commissioned, externally peer-reviewed.
